# Retraction: Knockdown of long non-coding RNA OIP5-AS1 suppresses cell proliferation and migration in ox-LDL-induced human vascular smooth muscle cells (hVMSCs) through targeting miR-152-3p/PAPPA axis

**DOI:** 10.1039/d1ra90047a

**Published:** 2021-01-27

**Authors:** Laura Fisher

**Affiliations:** Royal Society of Chemistry Thomas Graham House, Science Park, Milton Road Cambridge CB4 0WF UK advances-rsc@rsc.org

## Abstract

Retraction of ‘Knockdown of long non-coding RNA OIP5-AS1 suppresses cell proliferation and migration in ox-LDL-induced human vascular smooth muscle cells (hVMSCs) through targeting miR-152-3p/PAPPA axis’ by Xiangya Yang *et al.*, *RSC Adv.*, 2019, **9**, 32499–32509, DOI: 10.1039/C9RA06614D.

The Royal Society of Chemistry hereby wholly retracts this *RSC Advances* article due to concerns with the reliability of the data. The images in the article were screened by an image integrity expert. All of the western blot bands are over-contrasted, have almost no visible background and may not be genuine. In particular, the appearance of the first band in the β-actin control panel of Fig 4C is inconsistent with the other bands and may have been added on. The same concern also applies to the last band in the β-actin control panel in Fig 6C. Furthermore, the western blots and many other features of the article were found to be unexpectedly similar to western blots and features in a number of other papers with no overlapping authors.

In addition, the paper was analysed by experts who fact-checked the identities of the described nucleotide sequence reagents,^1^ and found errors with the following nucleotide sequence reagents reported in the article. The miR-152 reverse primer described in this article is not a reverse RT-PCR primer specific for hsa-miR-152, but a universal reverse primer. This paper therefore reports a wrongly described reagent, which impacts on the reliability of the results and may be misleading for future studies.

The authors were asked to provide the raw data for this article, but did not respond. Given the significance of the concerns about the validity of the data, and the lack of raw data, the findings presented in this paper are not reliable.

The authors have been informed but have not responded to any correspondence regarding the retraction.

Signed: Laura Fisher, Executive Editor, *RSC Advances*

Date: 15^th^ January 2021

## Supplementary Material
